# Exploring hot spots of short birth intervals and associated factors using a nationally representative survey in Bangladesh

**DOI:** 10.1038/s41598-022-13193-2

**Published:** 2022-06-09

**Authors:** Mohammad Zahidul Islam, M. Mofizul Islam, Md. Mostafizur Rahman, Md. Nuruzzaman Khan

**Affiliations:** 1 Department of Population Science, Jatiya Kabi Kazi Nazrul Islam University, Trishal, Mymensingh, Bangladesh; 2 Department of Public Health, La Trobe University, Melbourne, Australia; 3 Department of Population Science and Human Resource Development, University of Rajshahi, Rajshahi, Bangladesh

**Keywords:** Risk factors, Medical research, Epidemiology

## Abstract

Short Birth Interval (SBI, defined as < 33 months interval between the two most recent births or < 24 months between one live birth to the next pregnancy) is a public health problem in most low- and lower-middle-income countries. Understanding geographic variations in SBI, particularly SBI hot spots and associated factors, may help intervene with tailored programs. This study identified the geographical hot spots of SBI in Bangladesh and the factors associated with them. We analyzed women’s data extracted from the 2017/18 Bangladesh Demographic and Health Survey and the healthcare facility data extracted from the 2017 Service Provision Assessment. SBI was the outcome variable, and it was defined as an interval between consecutive births of 33 months or less, as recommended by the World Health Organization. The characteristics of mothers and their partners were the explanatory variables. Moran’s I was used to examine the spatial variation of SBI in Bangladesh whereas the Getis-Ord ${G}_{i}^{*}$(d) was used to determine the hot spots of SBI. The Geographical Weighted Regression (GWR) was used to assess the predictors of SBI at the enumeration areas’ level. The variables included in the GWR were selected using the exploratory regression and ordinary least square regression model. Data of 5941 women were included in the analyses. Around 26% of the total births in Bangladesh had occurred in short intervals. A majority of the SBI hot spots were found in the Sylhet division, and almost all SBI cold spots were in the Rajshahi and Khulna divisions. No engagement with formal income-generating activities, high maternal parity, and history of experiencing the death of a child were significantly associated with SBI in the Sylhet division. Women’s age of 34 years or less at the first birth was a protective factor of SBI in the Rajshahi and Khulna divisions. The prevalence of SBI in Bangladesh is highly clustered in the Sylhet division. We recommend introducing tailored reproductive health care services in the hot spots instead of the existing uniform approach across the country.

## Introduction

Despite the tremendous strides in improving maternal and child health, particularly in the Millennium Development Goals period of 2000–2015, maternal and child morbidity and mortality continue to be a major problem in low- and lower-middle-income countries (LMICs). Globally, almost 300,000 women die annually from causes related to pregnancy and childbirth, and 94% of these occur in LMICs^1^. Also, over 80% of 5.2 million global under-five mortality occurs in LMICs, although they only account for 52% of the global under-five population^2^. Around half of these deaths occur within the first 28 days of children’s lives and an additional 1.5 million occur within 1–11 months of birth^2^. Anemia, placental abruption, placenta previa, uterine rupture, preterm birth, low birth weight and congenital malformations are some of the dominant causes of many of these deaths, and they are preventable^1–4^. Inadequate birth spacing is incontrovertibly linked to many of these adverse health outcomes^5,6^. The reason is the lack of sufficient time to return to the normal pregnancy metabolic state before the next pregnancy and its effects on women’s nutritional, physical and emotional health^7^. Moreover, pregnancy in short intervals decreases maternal foetal concentrations, especially during the second and third trimesters, which can weaken connective tissue by preventing collagen cross-linking, thus increasing the risk of adverse pregnancy outcomes^8^. The World Health Organization recommends this interval to be at least 33 months. A shorter duration than this is identified as a short birth interval (SBI)^9^. Around one-fourth of pregnancies that end with live births in LMICs occur in SBIs^10–12^.

Previous research in LMICs primarily focused on sociodemographic risk factors of SBI, including the characteristics of mothers, children, husbands and other members of the households^10–14^. Participants’ geographical locations can also have impacts, mainly through modification of other risk factors of SBI^15^. The reason is that in LMICs, socio-economically advantaged and disadvantaged people usually live in clusters, therefore their distributions are not homogeneous across clusters in an area. As a result, risk factors of SBIs, such as women’s education, partner’s education, wealth quintile, could be different between various clusters in an area which further contribute to the regional variability of SBI^13,14,16^. However, while area-level differences in SBI rates were sometimes reported in previous research in LMICs, often these were measured across border geographical locations such as urban/rural and administrative divisions^10–14^. This overall estimate masks the local-level variation and, thereby, limits the ability to formulate policies and programs targeting specific areas or segments of people where much-needed public health interventions are needed^15^. Consequently, in many LMICs, we see mismatches between service requirements and service availability and misuse of limited but valuable resources. This is particularly true for Bangladesh, where healthcare policies and programs are usually adopted nationally. This approach directly or indirectly considers a uniform situation across the country and does not account for local-level needs. In this study, using two nationally representative surveys of Bangladesh, we aimed to determine the geographical distribution of SBI, explore the hot and cold spots of SBI and area-specific risk factors of SBI.

## Methods

This study draws on data from the 2017/18 Bangladesh Demographic and Health Survey (BDHS), which is a nationally representative data source and provides estimates of reproductive health, maternal and child health^17^. The methods of data collection and data collection procedure were reviewed and approved by the National Research Ethics Committee of the Bangladesh Medical Research Council. Informed consent was obtained from all participants. The survey is part of the Demographic and Health Survey (DHS) Program conducted in 90 LMICs. In Bangladesh, the Ministry of Health and Family Welfare supervised the survey and its partner organizations, The National Institute of Population Research and Training along with Mitra and Associates (an independent research firm) implemented this survey at the field level. Several development partners, including United Nations Population Fund (UNFPA) and United Nations Development Programme, provided financial support for this survey.

**Figure 1:**
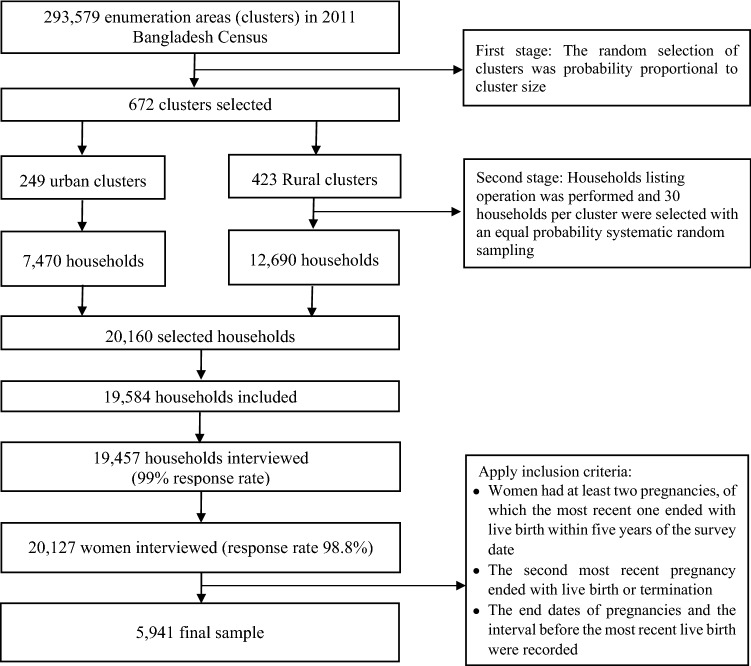
Flow chart of the study participants selected from the Bangladesh Demographic and Health Survey, 2017–18.

Following a two-stage stratified random sampling approach, the survey collected data from women of 15–49 years old living in the selected households. At the first stage of sampling, the survey selected 675 Enumeration Areas (EAs or clusters) covering urban and rural areas as well as eight administrative divisions of Bangladesh. These clusters were selected randomly from a list of 293,579 EAs or clusters, which were used as the sampling stratum for the survey. These clusters (from here the term “cluster” will be used) were created by the Bangladesh Bureau of Statistics as part of the most recent population Census in 2011. The household listing operation was conducted at the second stage of sampling and 30 households were selected from each cluster by systematic random sampling. A total of 20,160 households were selected, of which data collection was undertaken in 19,457 households with over 96% inclusion rate. There were 20,376 eligible women in the selected households. Of them, data were collected from 20,127 women with a response rate of 98.8%. The survey recorded birth interval data if mothers met the following criteria: (i) the woman had at least two pregnancies, of which the most recent one ended with live birth within five years of the survey date, (ii) the second most recent pregnancy ended with live birth or termination, and (ii) the end dates of pregnancies and the interval before the most recent live birth were recorded (Fig. 1). As such, these were inclusion criteria for this study.

The survey also collected the geographical location of each cluster using the Global Positioning System (GPS). The GPS reading was made at the center of each cluster, while efforts were made to ensure adequate satellite signal strength. For this, the data collectors ensured that they were not near any tall building or under any big tree. The points recorded were then randomly displaced to 5 kms in the rural area and 2 kms in the urban area. The DHS recorded those displaced cluster points in a shapefile (geographical data file) and released it along with the survey data.

We also used geographical data from the 2017 Bangladesh Health Facility Survey (BHFS), a nationally representative survey of healthcare facilities. This dataset includes 1524 healthcare facilities selected randomly throughout the country covering primary, secondary and tertiary level healthcare facilities. A detailed description of the sampling procedure of both surveys has been published in their survey reports^17,18^.

### Outcome variable

The outcome variable is SBI, defined as an interval of < 33 months between the two most recent births. The BDHS recorded this data in months by subtracting the date of birth of the most recent child to the date of birth or termination of the second most recent child. These dates were collected from the birth registration reports or immunization cards. If these were not available, mothers were requested to recall their memories. These women were referred to memorable events like the national or local election, floods or other natural disasters to help them recall their memories to estimate the accurate date of births.

### Explanatory variable

The explanatory variables were identified through a comprehensive literature search in the following five databases: Medline, Embase, Web of Science, CINHAL, and Google Scholar. A pre-designed search strategy was used with relevant keywords, including birth interval, birth spacing, and short birth interval. To identify the key factors, special attention was paid to the five studies conducted in Bangladesh^13,14,19–21^ and the studies conducted in some other LMICs^10–12^. The factors were age at birth of the most recent child (≤ 19, 20–34, ≥ 35), age at first birth (≤ 19, 20–34, ≥ 35), educational status of women and their husbands (no education, primary, secondary, higher), and women’s employment status (employed, not employed), sex of households’ head (male, female), women’s exposure to mass media (little exposed, moderately exposed, highly exposed) and the number of children ever given birth (≤ 2, > 2). Survival (yes *vs* no) of the second most recent child was also considered. The BDHS recorded these data in their main survey along with SBI data. The average distances from respondents’ houses to the nearest healthcare facilities that offer reproductive healthcare services were also considered an explanatory variable. The average Euclidean distance was calculated at the divisional level using the administrative boundary link method based on the geographical variables of the 2017/18 BDHS and 2017 BHFS datasets^22^. We used a regional-level average instead of the actual distance between the clusters and nearest health facilities because the BHFS survey included a sample for all health facilities except district-level health facilities and maternal and child welfare centres. Thus, a cluster's nearest health facilities might not have been selected and included in the survey, and hence the actual distance would be problematic. The details of these computation procedures can be found elsewhere^23^.

### Statistical analysis

The prevalences of SBI and the explanatory variables were estimated across clusters. Using the birth interval data recorded in the survey, we have calculated the prevalence of SBI for each cluster included in the BDHS with the standard formula: $\frac{\text{Number of eligible women reported SBI in a cluster }}{\text{Total eligible women in that particular cluster}}$. Using this formula, we identified the prevalence of women who experienced SBI for each cluster. So, the prevalence of all 672 clusters constituted a continuous variable, which was found to be normally distributed and suitable for using ordinary least square regression (OLS). A similar process was applied to calculate the prevalences of explanatory variables. They were also found normally distributed. A weighted sample was used to generate the prevalences of explanatory and outcome variables. This was done using the *svy* command available in STATA. The estimated cluster’s prevalences for explanatory and outcome variables were then merged with the GPS locations for clusters. This generated a data set containing prevalences of explanatory and outcome variables for 673 clusters distributed across Bangladesh. We then used this data to examine whether any geographical difference persists in the distribution of SBI in Bangladesh. For this, the hot spot analysis was conducted which followed three procedures as discussed elsewhere^24,25^. These were the Global Moran’s I statistic, Incremental Spatial Autocorrelation and the Getis-Ord Gi* statistic^24,25^. A False Discovery Rate correction method was applied to account for multiple and spatial dependence tests in Local Statistics of Spatial Association^23^. The importance of this correction method in DHS data has been described elsewhere^23,26^. Statistical significance was determined based on the z scores and p-values returned while running hot spot analysis.

We ran Ordinary Least Square (OLS) to identify the predictors of observed spatial patterns of SBI in Bangladesh. We checked the model assumptions for OLS and multicollinearity^27,28^. For this, the variables included in the OLS were first determined carefully by using exploratory regression, a data mining tool was used to select the variables as Stepwise Regressions do^29^. The exploratory regression model identifies the variables to be included in the OLS and the variables included in the OLS model meet all of its assumptions. They are, (i) coefficients of explanatory variables in a properly specified OLS model should be statistically significant and have either a positive or negative sign, (ii) there should not be redundancy among explanatory variables (free from multicollinearity), (iii) the model should be unbiased (heteroscedasticity or non-stationarity), (iv) the residuals should be normally distributed and revealed no spatial patterns, (v) the model should include key explanatory variables, and (vi) the residuals must be free from spatial autocorrelation. We also standardized all explanatory variables.

The OLS fits a linear regression to all of the data in the study area. Therefore, it did not answer the questions, (i) why clustering (if any) of SBI occurs in Bangladesh? and (ii) what factors are associated with the observed clustering? It is also important to know whether the relationships between the outcome variables and explanatory variables vary across areas and which explanatory variables show substantial influence. The Geographically weighted regression (GWR) answers these questions. We ran GWR with the variables that met the assumptions of the OLS model, as recommended in the previous studies^30,31^. Unlike other regression models that produce an overall estimate for the entire area, this model produces estimates of SBI’s determinants for each cluster^31^. Therefore, the findings reported are more specific to the clusters level. Statistical software Stata version 15.1 (Stata Corp, College Station, Texas, USA) was used to describe women’s characteristics, the differences in SBI across places and regions of residence and weighted proportions of outcome and explanatory variables. The ArcGIS version 10.6.1 (ESRI. ArcGIS Desktop: Release 10. Redlands, CA: Environmental Systems Research Institute. 2011) was used to perform geographical analyses, including hot spots and cold slots analysis, exploratory regression analysis, OLS and GWR. All methods were performed in accordance with the relevant guidelines and regulations.

## Results

### Background characteristics of the respondents

This study includes data of 5941 women who came from 672 clusters in the 2017/18 BDHS. The crude and age-standardized characteristics of the study sample are shown in Table 1. The average age of participants at their most recent births was 25.93 years (SD ± 5.13). On average, they received 6.12 years of education (SD ± 3.70) and gave birth to 2.85 (± 1.18) children. More than a quarter of the total live births occurred in SBI (26.26%).

**Table 1:** Characteristics of the respondents (N = 5941).

Characteristics	Crude estimate	Age-standardised estimate^a^
Women age at most recent child, mean years (± SD)	25.93 (5.13)	–
Women age at the first birth (± SD)	18.14 (3.10)	–
Women’s education, mean years (± SD)	6.12 (3.70)	6.47 (3.73)
Children ever born, mean number (± SD)	2.85 (1.18)	2.13 (1.26)
Mother engaged in a formal job, prevalence (95% CI)	46.81 (44.39–49.24)	44.0 (41.98–47.12)
Female sex of the household’s head	13.91 (12.55–15.39)	13.20 (11.98–15.30)
Women highly exposed to mass media	48.21 (45.57–50.86)	47.83 (45.66–49.59)
The child born from the second most pregnancy died	8.12 (7.20–9.15)	8.04 (7.23–9.13)
**Birth interval**		
Prevalence of short birth interval (95% CI)	26.26 (24.84–27.94)	25.6 (22.9–28.8)
Prevalence of non-short birth interval (95% CI)	73.64 (72.06–75.16)	74.0 (41.2–75.8)

^a^Age-standardisation was performed using the age structure of women of 15–49 years included in the 2011 Bangladesh National Census.

### Geographical distribution of the prevalence of short birth interval

The geographical distribution of the prevalence of SBI in Bangladesh is presented in Table 2. We found a statistically significant difference in SBI prevalence across the places of residence and divisions. The prevalence of SBI in rural areas was around 27%, compared to 24% in urban areas. Among the eight administrative divisions, the Sylhet division sits on the top of the league table (46%), and the Khulna division sits on the bottom (19.99%).

**Table 2:** Number of births with short birth intervals (weighted prevalence) in Bangladesh by place of residence and administrative division, BDHS 2017/18 (N = 5941).

	Short birth interval (weighted)		Chi-square test
	Yes (n = 1566)	No (n = 4398)	
**Place of residence**			
Urban	1191 (75.66)	3207 (24.34)	*p* < 0.05
Rural	375 (72.93)	1191 (27.07)	
**Division**			
Barishal	72 (21.46)	262 (78.54)	*p* < 0.01
Chattogram	379 (28.86)	933 (71.14)	
Dhaka	352 (24.02)	1114 (75.98)	
Khulna	100 (19.99)	402 (80.01)	
Mymensingh	142 (28.08)	365 (71.92)	
Rajshahi	137 (21.39)	505 (78.61)	
Rangpur	130 (20.72)	496 (79.28)	
Sylhet	254 (45.99)	298 (54.01)	

Numbers within the parenthesis are weighted prevalence.

### Hot spots and cold spots of short birth interval in Bangladesh

**Figure 2:**
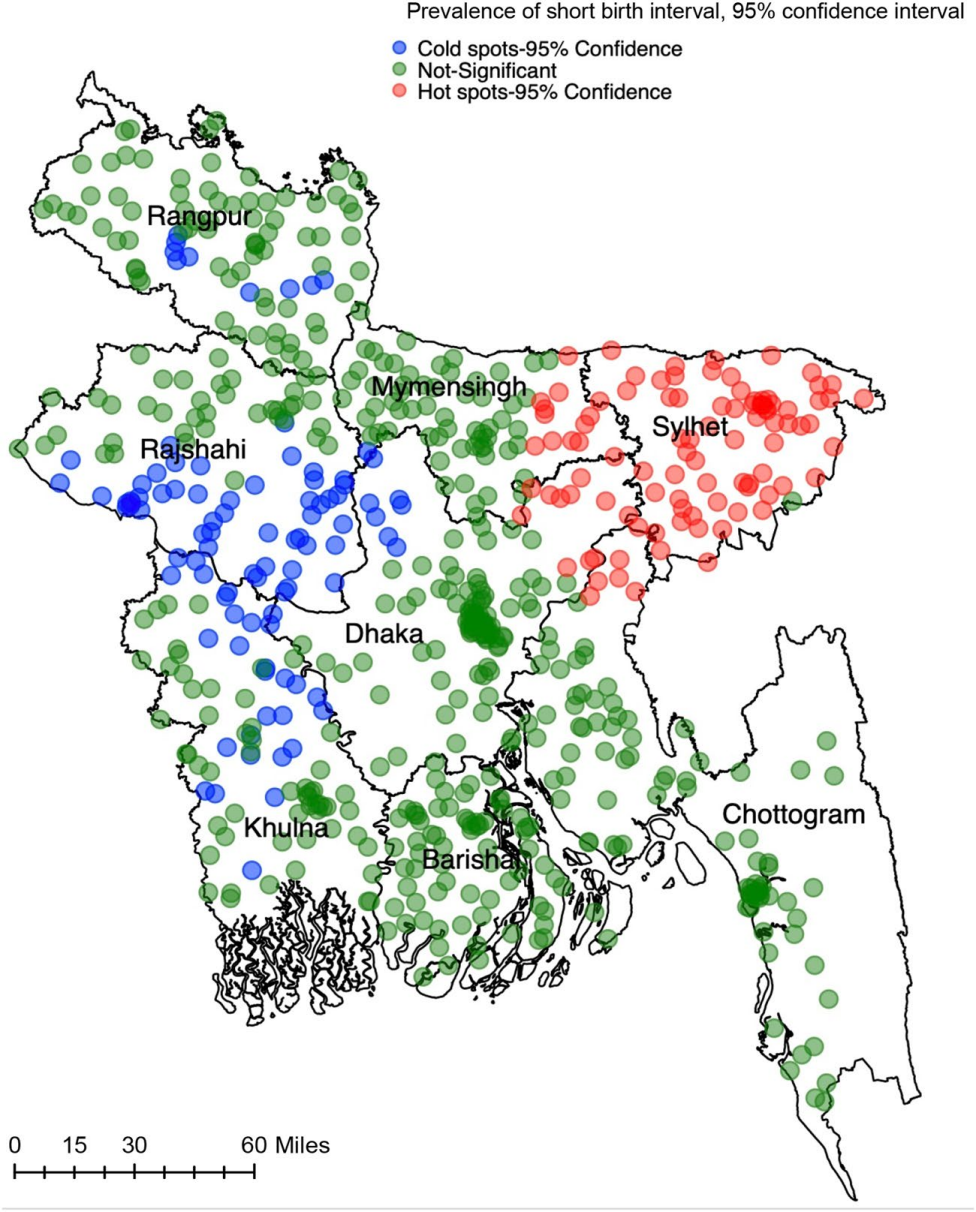
Hot spots and cold spots of short birth intervals in Bangladesh. Map created using ArcGIS version 10.6.1 (ESRI. ArcGIS Desktop: Release 10. Redlands, CA: Environmental Systems Research Institute, 2011).

We found evidence of statistically significant clustering of SBI in the study area (Moran’s I = 0.330590, *p* < 0.01). The Getis-Ord G statistic revealed the high clustering across clusters (*p* < 0.01) (Fig. 2). A relatively high number of SBI hot spots were found in the Sylhet division, and SBI cold spots were found in parts of the Rajshahi and Khulna divisions.

### Model comparisons: OLS and GWR

The results of the OLS model are presented in Table 3. The results demonstrate that five explanatory variables had a positive relationship with SBI. None of the variables had multicollinearity. The adjusted R^2^ was 0.62. The Akaike information criterion was − 988.13.

**Table 3:** Ordinary least square regression model identifying significant factors of short birth interval in Bangladesh, BDHS 2017/18.

Variable category	Coefficient	Standard error	t-statistics	Probability	Robust standard-error	Robust t-statistics	Robust probability	VIF
Women’s age at first birth, ≤ 19 years	1.05	0.09	32.24	< 0.01	0.05	36.14	< 0.01	3.40
Women’s age at first birth, 20–34 years	0.92	0.06	29.12	< 0.01	0.03	34.12	< 0.01	1.95
Husbands did not receive formal education	1.13	0.23	41.12	< 0.01	0.07	42.34	< 0.01	3.12
Women not engaged in formal work	0.78	0.12	23.17	< 0.01	0.06	25.12	< 0.01	2.98
Gave birth to three or more children	0.34	0.07	21.12	< 0.01	0.03	14.12	< 0.01	1.93
The child born from the second most pregnancy died	0.41	0.06	7.00	< 0.01	0.04	6.00	< 0.01	1.40
Intercept	0.11	0.02	2.97	< 0.01	0.1	1.94	< 0.01	–
**Model diagnostics**								
Number of observation (clusters)	672	Akaike’s Information Criterion (AIC)					− 988.13	
Multiple R-squared	0.62	Adjusted R-squared					0.62	
Joint F-Statistics	1824.14	Probability (> F), (6672) degrees					< 0.01	
Joint Wald Statistics	158.13	Probability (> chi-squared), (6) degrees of freedom					< 0.01	
Koenker (BP) Statistics	1892.14	Probability (> chi-squared), (6) degrees of freedom					< 0.01	
Jarque–Bera Statistics	0.86	Probability (> chi-squared), (6) degrees of freedom					< 0.01	

The effects of the five variables selected for SBI hot spots and cold spots in the area level were determined using the GWR. The summary results of this model fit are presented in Table 4. Model fitness was improved with the GWR over the OLS. The Akaike information criterion value was − 988.13 for the OLS model and—1024.23 for the GWR model. The reported adjusted R-squared for the GWR model was 0.65, 3% higher than the OLS model.

**Table 4:** Geographically weighted regression model assessing factors of short birth interval in Bangladesh, BDHS 2017/18.

Explanatory variables	Women’s age at first birth, ≤ 19 years, Women’s age at first birth, 20–34 years, Husbands did not receive formal education, Women not engaged in formal work, Gave birth to three or more children, Experienced death of a child
Residual squares	17.87
Effective number	81.24
Sigma	0.18
Akaike information criterion	− 1024.23
Multiple R-squared	0.67
Adjusted R-squared	0.65

### Predictors of short birth interval: hot spots and cold spots

**Figure 3:**
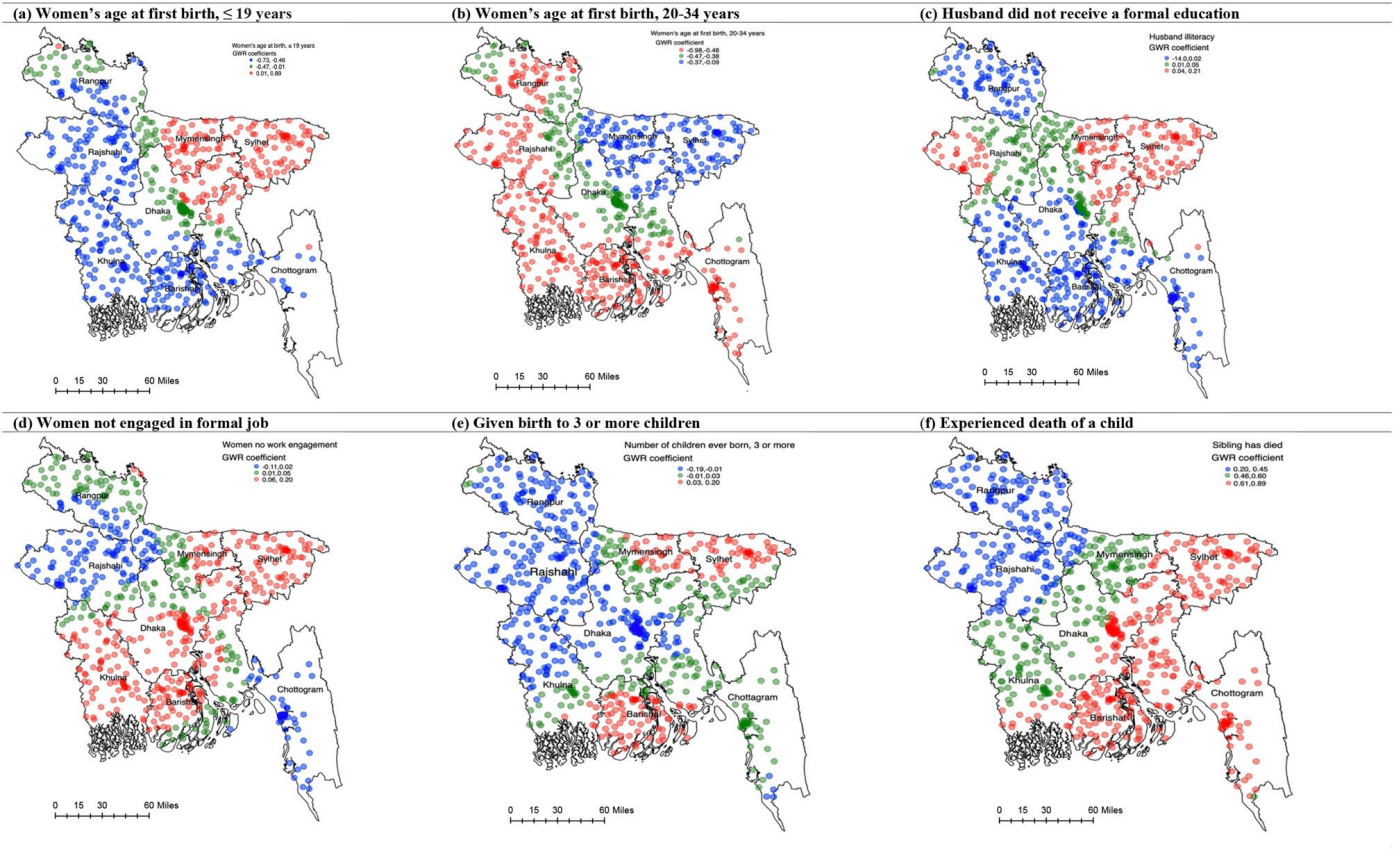
(**a**–**f**) GWR coefficients predicting short birth intervals in Bangladesh, BDHS 2017/18.

The cluster-wise coefficients of the GWR model are plotted in Fig. 3a–f. The significant predictors of SBI in the Sylhet division, where a majority of the SBI hot spots are located, were no formal education of husbands (Fig. 3c), women doing no formal jobs (Fig. 3d), having three or more children (Fig. 3e), and experiencing the death of a child (Fig. 3f). On the contrary, in the Rajshahi and Khulna divisions where most of the SBI cold spots were located, maternal age of 34 years or less at the first birth (Fig. 3b) was a significant protector of SBI.

## Discussion

This study provides evidence that along with the socio-demographic factors known to be associated with a high prevalence of SBI in Bangladesh^14,16,20^, area-level variations are also important. This is the first study in the Bangladesh context that explored the factors determining such area level variations, including factors responsible for SBI hot spots and cold spots. Unemployed women, those who gave birth to three or more children, experienced the death of a child, or whose husbands received no formal education were significantly more likely than others to be located in SBI hot spots. Women who gave their first birth at the age of 19 years or earlier and 20–34 years were significantly more likely to be living in SBI cold spots. These findings should be used to design future policies and programs in order to reduce prevalence of SBI and its associated adverse outcomes.

The observed prevalence of SBI (26%) is consistent with the results of a nationally representative study conducted recently in Bangladesh^16^ and in the range of SBI (19–66%) reported in LMICs^32,33^. This study also found a relatively high prevalence of SBI in the Sylhet division, where a majority of SBI hot spots are located. On the other hand, SBI cold spots are mainly located in parts of the Rajshahi and Khulna divisions. This is a new observation for Bangladesh, although the divisional level variations in SBI have been reported in previous studies^13,14,16^. These divisional variations in SBI hot spots and cold spots are due to the division-level variations in socio-demographic and cultural characteristics of women and their partners and their perceptions regarding the desired number of children.

Previous studies in Bangladesh consistently reported high rates of early marriage, relatively low age at first birth, and low rates of formal education in the Sylhet division^34,35^. These characteristics, both individually and together, can affect SBI. Our results also suggest that these factors are the significant predictors of SBI in the SBI hot spots area in the Sylhet division. A possible reason for such association is that couples with these characteristics are less likely to access maternal healthcare services, including intrapartum, birthing, and post-partum care^36–38^. Moreover, in the current form of maternal healthcare services delivery in Bangladesh, post-partum care visit on the fourth week of the live birth is dedicated to providing counselling regarding family planning and contraception^39^. This approach does not help increase family planning and contraception services because post-partum care visits at the fourth week of live birth are still very low in Bangladesh^39^. Indeed, many women in Bangladesh have a misapprehension that once a live birth has occurred, the issue of pregnancy is over, and it is unnecessary to visit a healthcare center for post-partum care, particularly at the fourth week of live birth. This tendency is even higher among women of disadvantaged backgrounds. Consequently, many women end up with another pregnancy in a short interval. Additionally, women with these characteristics are less likely to receive family planning counselling which is offered at the household level by family planning workers^40^.

Although the underlying reasons for such low use of services in the Sylhet division have yet not been explored, we believe this is mainly due to inadequate knowledge of reproductive goals^40^. Moreover, there are studies in Bangladesh, including the Sylhet division, that found women of disadvantaged backgrounds are highly influenced by religious misconceptions. For instance, many couples believe that the religion Islam (the religion of over 90% of the population in Bangladesh) supports *taking children as many as they want, and* contraception use is comparable to the *killing of humans*^40–42^. Consequently, the current approach to family planning services, including visits to women’s homes by family planning workers every 14 days to provide reproductive counselling and contraception, may not work effectively in this division. Indeed, several recent studies reported a high prevalence of unmet need for contraception and particularly modern contraception in Sylhet compared to the other divisions^43,44^. Also, the prevalence of unintended pregnancy in this division is higher than in other parts of Bangladesh^40,45^, and most of them occur in shorter intervals of the previous births^16^. Also, a relatively high prevalence of men in the Sylhet division is either migrated aboard or locally^46^. Women having migrated partners are less likely to receive maternal healthcare services, a finding reported in Nepal^47^ and Bangladesh^48^. Consequently, they have inadequate knowledge regarding birth spacing.

Literature suggests that the prevalence of adverse pregnancy outcomes, including child mortality, is relatively high in the Sylhet division and low in the Rajshahi and Khulna divisions^49,50^ and are aligned with the SBI hot spots and cold spots, respectively. There seems to be a two-way relationship between adverse pregnancy outcomes and SBI; adverse outcomes occur due to a relatively high number of births in shorter intervals and vice versa. Findings from the studies in other settings of LIMCs^51–53^ demonstrate relatively long birth intervals among couples with fewer children. Couples experiencing the death of a child or even witnessing such an event among the neighbours, are usually motivated to take another child considering the uncertainty, often in a shorter interval^52^. Similarly, women who are not engaged in formal jobs are likely to have babies in short intervals^15^.

The findings of this study highlight the need for tailored programs in Bangladesh in general and the Sylhet division in particular to reduce the prevalence of SBI. Strengthening reproductive healthcare service delivery, including intrapartum, delivery, postpartum, and postpartum contraceptive services should be prioritized. Providing integrated reproductive healthcare services may help improve the current service delivery. Also, tailoring service modality considering the divisional level barriers is needed^39^, as it is not possible in the current uniform top-down policy approach^38,39^.

As far we know, this is the first study that explored the hot spots and cold spots of SBI and its associated factors in Bangladesh. The explanatory variables considered in this study were chosen based on a comprehensive review of the existing literature and finally by following the proper statistical model building techniques. The data were collected from two nationally representative surveys conducted in the same year using validated questionnaires. However, the analysis of cross-sectional data means that we are unable to establish temporality. To ensure the privacy of the respondents, the BDHS displaced clusters’ locations that we used in plotting our results in maps, up to 5 kms in rural and 2 kms in urban areas. Thus, the areas plotted in the maps as SBI hot spots or cold spots are slightly different from the actual areas from where data were collected, although divisions of data collection were the same. Moreover, distance bands used will have an impact on the results, such as hot spots identified. However, the findings are still valid as our results only highlight the potential areas of SBI hot spots or cold spots. Another limitation was the scales at which relationships between explanatory factors and SBI were considered. The GWR model considers a single bandwidth, i.e., cluster-wise average value (prevalence) of explanatory and outcome variables as per this study^54^. However, prevalences can vary across several parts within a cluster that can be addressed using a multi-scale GWR model. Bangladesh is a highly densely populated country (1265 persons per square kilometre), and 30 households that BDHS included from each cluster are usually located within a very small geographical area. For such dense geographical locations (bandwidth), the GWR fits reasonably^55^, and the estimates are unlikely to be affected by the presence of collinearity (both wider and local level) if the study sample size is ≥ 1000. Our study sample is around six times larger (N = 5941)^56^. Therefore, our findings are still valid although further exploration should be performed using a multiscale approach once supportive geographical data are available. Further, the OLS in GWR cannot account for the overdispersion terms—a feature that is not yet available in GWR. Moreover, besides the socio-demographic factors included in this study, area level and environmental factors and ecological nature could also be important predictors of SBI hot spots and cold spots in Bangladesh, but we could not consider those variables in our analysis as they were not available. Evidence of the effects of health facility level factors such as preparedness of the nearest health facility to provide modern contraception and distance between households and health centres are found as important predictors of SBI in another study of this series^16^. However, since they are common across the entire cluster, they are unlikely to exhibit sufficient variability at the local level required for GWR, therefore they are not included in this study. In addition, GWR is not being able to incorporate unit-level spatial random effects, which may lead to the observed spatially varying coefficients*.* Moreover, we did not include the distance variable in the model as it could confound GWR results that employ distance-based analyses^57^. However, our adjusted variables explained around 65% of the total occurrences of SBI.

## Conclusion

We found evidence of substantial geographical variations in SBI in Bangladesh. SBI hot spots are mainly located in the Sylhet division, and SBI cold spots are mainly located in parts of the Rajshahi and Khulna divisions. Divisional variations in socio-demographic characteristics of women and their partners could be the main reasons for such geographical variation in SBI hot spots and cold spots. Targeted and divisional level policies and programs to provide integrated intrapartum, birthing, and postpartum care, including postpartum contraception, are needed to reduce the prevalence of SBI in Bangladesh in general and in the Sylhet division in particular.

## Data Availability

The datasets used and analyzed in this study are available from the Measure DHS website: https://dhsprogram.com/data/available-datasets.cfm.
